# Characterizing *zonulin* and *par2* Expression in Zonulin Transgenic and Zonulin Inhibition Mouse Models of Motility and Inflammation

**DOI:** 10.3390/ijms26136381

**Published:** 2025-07-02

**Authors:** Enid E. Martinez, Jordan D. Philpott, Jinggang Lan, K. Marco Rodriguez Hovnanian, Alessio Fasano

**Affiliations:** 1 Department of Anesthesiology, Critical Care and Pain Medicine, Boston Children’s Hospital, Boston, MA 02115, USA; enid.martinez@childrens.harvard.edu (E.E.M.); jphilpott@mgh.harvard.edu (J.D.P.);; 2 Mucosal Immunology and Biology Research Center, Department of Pediatrics, Massachusetts General Brigham for Children, Boston, MA 02129, USA; 3 Harvard Medical School, Harvard University, Boston, MA 02115, USA; 4 Department of Pediatrics, Division of Pediatric Gastroenterology and Nutrition, Mass General Brigham for Children, Boston, MA 02114, USA

**Keywords:** gastrointestinal (GI), motility, transit, zonulin, protease-activated receptor 2 (PAR2), inflammation, mouse model

## Abstract

We aimed to examine the effect of zonulin and zonulin inhibition on gastrointestinal (GI) motility and the mRNA expression of zonulin and the protease-activated receptor 2 (*par2*), the primary receptor for zonulin, under conditions of inflammation by lipopolysaccharide (LPS) injection. The experimental models included zonulin transgenic mice (*ztm*), *par2* knockout *ztm* (*ztm-par2 −/−*), *ztm* exposed to the zonulin inhibitor AT1001 (*ztm*-AT1001), and wildtype mouse controls. GI transit was measured by fluorescein isothiocyanate-dextran and mRNA expression by real-time quantitative polymerase chain reaction in whole, and in epithelial and non-epithelial tissues of all GI segments. There were no differences in the GI transit between mouse groups at baseline. After the LPS injection, *ztm* mice had an attenuated slowing of the GI transit compared to wildtype mice. The zonulin-inhibited mice had motility patterns similar to wildtype mice. zonulin upregulation was noted in GI segments of the *ztm*, *ztm-par2 −/−,* and *ztm-*AT1001 after the LPS injection. Differences in motility patterns between *ztm* and zonulin inhibition models despite zonulin expression in GI segments of all mouse groups supports that PAR2 is key for zonulin’s effect on motility under conditions of inflammation. However, the findings from the epithelial and non-epithelial compartments suggest that the pathway of activity is complex and likely indirect.

## 1. Introduction

The epithelial barrier lining the gastrointestinal (GI) tract plays a vital role in maintaining physiological homeostasis by regulating bidirectional signaling across the barrier. Gastrointestinal functions such as nutrient absorption, energy regulation, immune tolerance, endocrine signaling and motility can all be impacted by changes in the transepithelial signaling of the GI tract [1,2,3,4,5]. Transepithelial trafficking increases, particularly under conditions of inflammation, thereby contributing to GI dysfunction under acute or chronic conditions of systemic inflammation [6,7,8,9,10]. Understanding the relationship between transepithelial barrier trafficking and other GI functions, such as motility, is key to restoring GI homeostasis when the epithelial barrier is disrupted, particularly under inflammatory conditions.

Zonulin is a protein that reversibly disrupts the epithelial barrier, allowing for an increase in transepithelial trafficking and thereby potentially impacting multiple GI functions beyond the epithelial barrier [11,12,13]. Zonulin is increased under conditions of local or systemic inflammation [14,15,16]. Increases in zonulin have been associated with multiple inflammatory conditions including obesity, celiac disease, critical illness and COVID-19-associated multisystem inflammatory syndrome [14,15,17,18,19]. Multiple aspects of GI homeostasis are impacted in these conditions, including motility, supporting a role for zonulin-mediated transepithelial trafficking in the development of dysmotility.

We have previously shown an association between systemic levels of zonulin and post-operative gastric dysmotility in a cohort of pediatric patients undergoing surgery and requiring critical care [19]. In a mouse model of inflammation, we demonstrated differences in the motility patterns in the small intestine of zonulin transgenic mice (ztm) compared to wildtype (wt) [19]. The mechanism by which zonulin is associated with dysmotility, however, is unclear. The primary receptor by which zonulin acts is the protease-activated receptor 2 (PAR2) [12]. Four PARs have been described, with the unique ability to self-activate by a protease-mediated cleavage of a tethered ligand or directly by external agonists [20,21]. PAR2 is present on diverse cell types throughout the GI tract in both the epithelial and non-epithelial layers, such as epithelial, immune and neuronal cells [20,22]. PAR2 activation by native and synthetic proteases in the GI tract has been found to impact GI motility by influencing intestinal contractility [23,24,25].

We have demonstrated that PAR2 is key for zonulin to exert its effect on the epithelial barrier; however, whether PAR2 also contributes to the effects of zonulin on GI motility is not clear. The effect of zonulin on motility may be direct or indirect. A direct mechanism may be that zonulin binds on PAR2 on key downstream cells in the non-epithelial compartment, such as neuronal or muscular cells that subsequently regulate and trigger contractility. An indirect mechanism may be that zonulin binds to PAR2 on epithelial cells on the epithelial compartment of the GI tract, driving transepithelial trafficking and the trafficking of microbial products, metabolites, etc., which impacts downstream cells that regulate motility such as enteroendocrine or immune cells.

To address this gap, this study aimed to examine the relationship between zonulin, PAR2 and motility under conditions of inflammation. Our approach included two models of zonulin inhibition. The first was employing a PAR2 knockout mouse and crossing it with *ztm*. The second mode of inhibition exposed *ztm* to AT1001, a zonulin inhibitor, prior to triggering inflammation [26]. zonulin and *par2* differences on whole tissue and epithelial and non-epithelial tissue from individual GI segments, among the diverse zonulin expressing mouse models, were examined using quantitative real-time PCR.

## 2. Results

### 2.1. GI Motility

**Figure 1:**
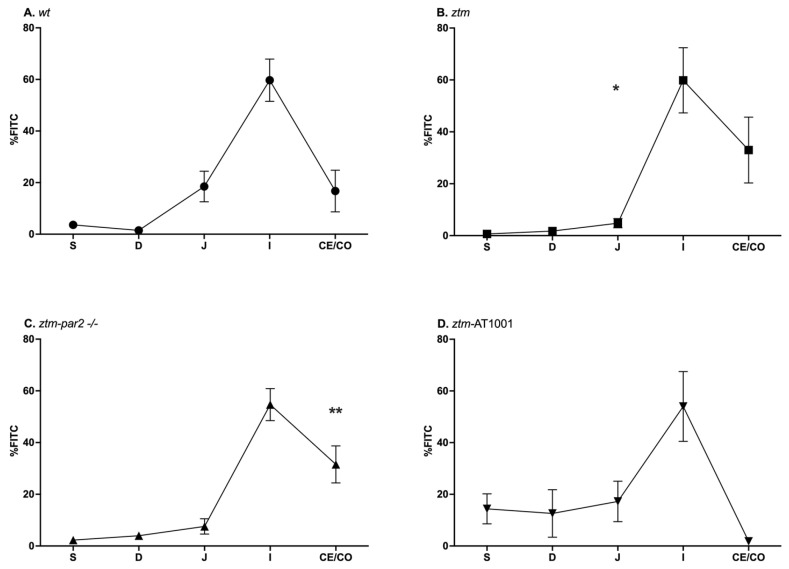
Gastrointestinal (GI) transit by FITC-dextran distribution in PBS-injected mice. Panel (**A**) depicts GI transit for wildtype (wt) mice (closed circles) which serves as control for statistical analysis of all experimental mouse groups; Panel (**B**) depicts GI transit for zonulin transgenic mice (*ztm*) (closed squares). *ztm* had less %FITC than (wt) in the jejunum, * *p*-value < 0.05; Panel (**C**) depicts GI transit for *ztm*-protease activated receptor 2 knockout *(ztm-par2 −/−)* mice (closed upward-facing triangle). *ztm-par2 −/−* had greater %FITC in the cecum/colon than (wt) ** *p*-value < 0.01: Panel (**D**) depicts GI transit for *ztm* exposed to AT1001 *(ztm-AT1001) (*downward-facing triangle). S: stomach; D: duodenum; J: jejunum; I: ileum; Ce: cecum; and Co: colon. Each symbol represents the mean %FITC for that segment and the standard mean error. *n* = 6–12, male and female mice; Dirichlet regression performed to compare %FITC between mouse groups and GI segments with (wt) serving as control.

GI motility was examined by the transit of FITC-dextran, with faster transit being represented by FITC-dextran reaching segments farther in the GI tract. Under baseline conditions, (wt), *ztm*, *ztm-par2 −/−* and *ztm*-AT1001 mice all had a similar pattern of FITC-dextran transit, with a peak %FITC in the ileum ((wt): 59.7 ± 27% FITC, *ztm*: 59.8 ± 35.6% FITC, *ztm-par2 −/−*: 54.7 ± 20.6% FITC, *ztm-AT1001*: 54 ± 33% FITC) (Figure 1). *ztm* and *ztm-par2 −/−* mice also had significant differences in %FITC in the jejunum and cecum/colon, respectively, compared to (wt), *(*jejunum*-ztm*: 4.8 ± 5% versus (vs) (wt): 18.5 ± 9.8% FITC, *p* = 0.04; cecum/colon-*ztm-par2 −/−*: 31.5 ± 23.8%, (wt): 16.7 ± 26.8%, *p* = 0.003) (Supplementary Table S1).

**Figure 2:**
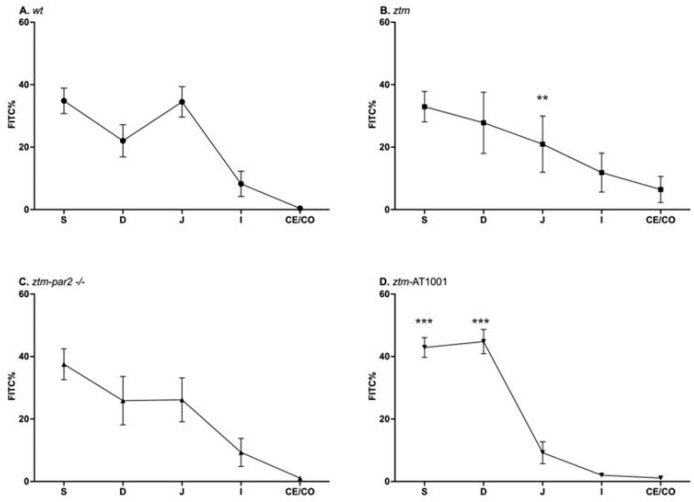
Gastrointestinal (GI) transit by FITC-dextran of LPS-injected mice. Panel (**A**) depicts GI transit for wildtype (wt) mice (open circles) which serves as control for statistical analysis of all experimental mouse groups; Panel (**B**) depicts GI transit for zonulin transgenic mice (*ztm*) (open squares). *ztm* had less %FITC than (wt) in the jejunum, ** *p*-value < 0.01; Panel (**C**) depicts GI transit for *ztm*-protease activated receptor 2 knockout (*ztm-par2 −/−*) mice (open upward-facing triangle): Panel (**D**) depicts GI transit for *ztm* exposed to AT1001 (*ztm-AT1001*) (open downward-facing triangle). *ztm*-AT1001 had more %FITC in the stomach and duodenum than (wt), *** *p*-value < 0.001. S: stomach; D: duodenum; J: jejunum; I: ileum; Ce: cecum; and Co: colon. Each symbol represents the mean %FITC for that segment and the standard mean error. *n* = 6–16, male and female mice; Dirichlet regression performed to compare %FITC between mouse groups and GI segments with (wt) serving as control.

Upon LPS exposure, all mouse groups had significant slowing in their transit, represented by the retention of FITC in the proximal segments of the GI tract, i.e., stomach and duodenum. The *ztm* had less %FITC in the jejunum (*ztm*: 21 ± 25.5%, (wt): 23.3 ± 20.5%) and more %FITC in the ileum, cecum, and colon than the (wt) control (ileum + cecum + colon: *ztm*: 18.3 ± 29.4%, (wt): 8.6% ± 16.2%), reflecting faster transit (Figure 2A,B). Inhibiting zonulin reduced the %FITC in the ileum, cecum, and colon in the *ztm-par2 −/−* and *ztm-*AT1001 compared to *ztm*, thereby reverting the phenotype back to the (wt) (ileum + cecum + colon: *ztm-par2 −/−*: 10.4 ± 13.9%, *ztm-*AT1001: 3.1% ± 2.1%, (wt): 8.6% ± 16.2%) (Figure 2C,D).

Analyzing these %FITC values between GI segments and mouse groups using a Dirichlet regression identified a lower %FITC in the jejunum of the *ztm* compared to (wt) [OR = 0.24, 95% CI (0.11, 0.54), *p* < 0.001] and a higher %FITC in the stomach and duodenum of *ztm*-AT1001 compared to (wt) [stomach-OR = 3.16, 95% CI (1.66, 5.99), *p* < 0.001; duodenum-OR = 5.75, 95% CI (3, 11.99), *p* < 0.001] (Supplementary Table S2).

### 2.2. zonulin mRNA Expression—Whole Tissue

**Figure 3:**
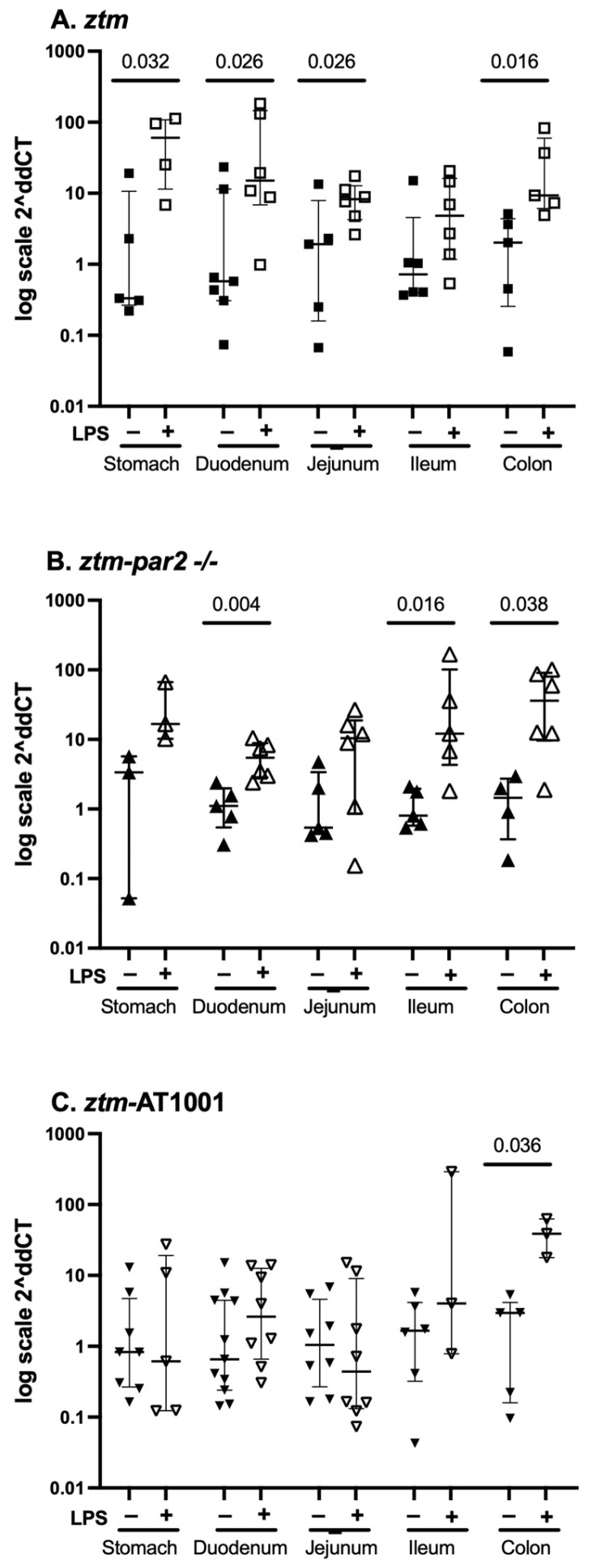
zonulin mRNA expression throughout the gastrointestinal tract. Panel (**A**) depicts data from PBS-(closed squares) and LPS (open squares)-injected zonulin-transgenic mice (*ztm*). LPS-injected *ztm* had more zonulin expression in the stomach, duodenum, jejunum and colon than PBS-injected *ztm, p* < 0.05; Panel (**B**) depicts data from PBS-(closed upward-facing triangles) and LPS (open upward-facing triangles)-injected protease-activated receptor 2 knockout *ztm* (*ztm-par2 −/−*) mice. LPS-injected *ztm-par2 −/−* had more zonulin expression in the duodenum, ileum and colon than PBS-injected *ztm-par2 −/−* mice, *p* < 0.05; Panel (**C**) depicts data from PBS-(closed downward-facing triangles) and LPS (open downward-facing triangles)-injected *ztm* exposed to AT1001 (*ztm*-AT1001). LPS-injected *ztm*-AT1001 mice had more zonulin expression in the colon than PBS-injected *ztm*-AT1001, *p* < 0.05. Data are summarized as median (25th, 75th); Kruskal–Wallis test controlled for multiple comparisons between mouse groups within each gastrointestinal tissue represented here, statistical significance set at *p*-value < 0.05. Data are presented as 2^−ΔΔ*CT*^ based on the delta cycle threshold (Ct) [dCt = target gene Ct − housekeeping gene Ct (18S)] of individual data − the mean of the PBS dCt within each mouse group.

We examined zonulin mRNA expression in all GI tissues of *ztm*, *ztm-par2 −/−*and *ztm*-AT1001 mice at baseline and under inflammatory conditions. zonulin mRNA expression was greater with LPS-injection compared to PBS in the stomach, duodenum, jejunum and colon of *ztm*, the duodenum, ileum and colon of the *ztm-par2 −/−* and the colon of *ztm-*AT1001 mice (Figure 3).

We also examined zonulin mRNA expression for each GI segment between mouse groups (e.g., zonulin mRNA expression in the stomach of *ztm*, *ztm-par2 −/−* and *ztm*-AT1001) and between each GI segment within individual mouse groups (e.g., stomach, duodenum, jejunum, etc., for *ztm*) (Table 1). Under baseline conditions, *ztm*-AT1001 had more zonulin mRNA expression in the duodenum than *ztm*, [median (25th, 75th) Ct zonulin*-18S*: *ztm* 18.36 (15.99, 21.52) versus *ztm-*AT1001 14.23 (12.07, 16.23), Kruskal–Wallis test *p* = 0.021], whereas the *ztm* had more zonulin mRNA expression than *ztm-par2 −/−* in the colon, [median (25th, 75th) Ct zonulin*-18S*: *ztm* 14.77 (13.12, 15.49) versus *ztm-par2 −/−* 17.59 (16.33, 18.08), Kruskal–Wallis test *p* = 0.013]. There were no differences in zonulin mRNA expression between mouse groups within each GI segment under inflammatory conditions (Table 1).

Differences in zonulin mRNA expression between tissues within mouse groups were noted under baseline conditions for *ztm* and *ztm-*AT1001 and under inflammatory conditions for *ztm-par2 −/−* (Table 1).

### 2.3. par2 mRNA Expression—Whole Tissue

**Figure 4:**
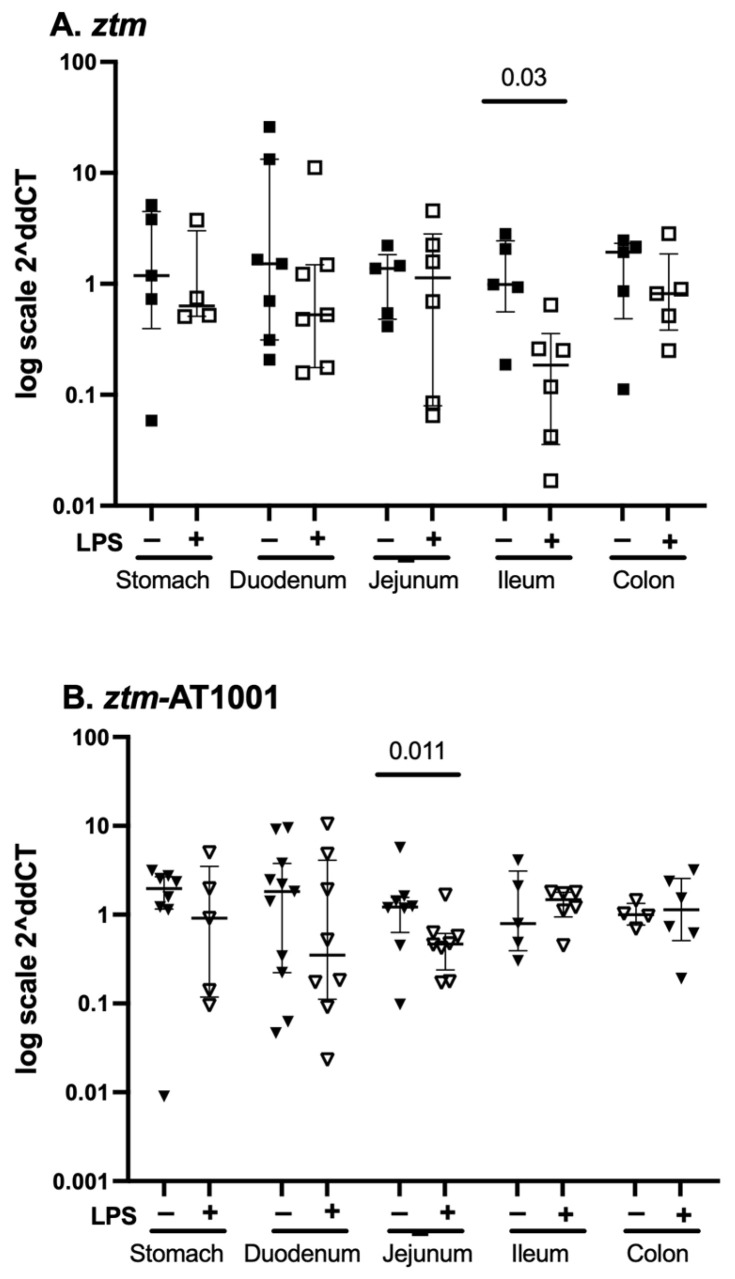
*par2* mRNA expression throughout the gastrointestinal tract. Panel (**A**) depicts data from PBS-(closed squares) and LPS (open squares)-injected zonulin transgenic mice (*ztm*). LPS-injected *ztm* had less *par2* expression in the ileum than PBS-injected *ztm*, *p* < 0.05. Panel (**B**) depicts data from PBS-(closed downward-facing triangles) and LPS (open downward-facing triangles)-injected, *ztm* exposed to AT1001 (*ztm*-AT1001). LPS-injected *ztm*-AT1001 had less *par2* expression in the jejunum than PBS-injected *ztm*-AT1001, *p* < 0.05. Data are summarized as median (25th, 75th); Mann–Whitney U test between mouse groups within gastrointestinal tissue. Data are presented as 2^−ΔΔ*CT*^ based on the delta cycle threshold (Ct) [dCt = target gene Ct − housekeeping gene Ct (18S)] of individual data − the mean of the PBS dCt within each mouse group.

Given that zonulin acts via PAR2 and that the *ztm-par2 −/−* versus *ztm*-AT1001 inhibition models demonstrated different motility patterns, we examined *par2* mRNA expression in *ztm* and *ztm*-AT1001 mice in all GI segments under baseline and inflammatory conditions. There was an overall pattern of *par2* mRNA expression downregulation under inflammatory conditions compared to the baseline for *ztm* and *ztm*-AT1001 in all GI segments (Figure 4). Downregulation after LPS-injection was statistically significant in the ileum of *ztm* [median (25th, 75th) Ct *par2-18S*: PBS-10.28 (8.99, 11.52) versus LPS-12.80 (11.88, 15.17), Mann–Whitney U Test *p* = 0.03; jejunum of the *ztm-*AT1001 [PBS 10.10 (9.74, 11.19) versus LPS 11.49 (11.09, 12.57), Mann–Whitney U test *p* = 0.011] (Figure 4). When comparing *par2* expression within GI segments between mouse groups, *ztm*-AT1001 mice had less *par2* mRNA expression in the ileum and colon and more in the jejunum than *ztm* under baseline conditions (Table 2). Under inflammatory conditions, *par2* mRNA expression was lower in the ileum and colon of *ztm*-AT1001 than *ztm* (Table 2). *ztm-*AT1001 had differences in *par2* mRNA expression in individual GI segments under both baseline and inflammatory conditions (Table 2).

### 2.4. zonulin mRNA Expression in Epithelial Versus Non-Epithelial Compartments

zonulin can be expressed in cells within the epithelial and non-epithelial compartments of the GI tract; therefore, we examined zonulin mRNA expression within these two compartments in all GI segments and zonulin-producing mouse groups. Table 3 and Table 4 depict median (25th, 75th) Ct zonulin*-18S* mRNA expression in the epithelial and non-epithelial compartments of *ztm*, *ztm-par2 −/−* and *ztm*-AT1001 under baseline and inflammatory conditions, respectively.

#### 2.4.1. Baseline Conditions

Median zonulin mRNA expression under baseline conditions for all mouse groups was greater in the non-epithelial tissues compared to the epithelial tissues [epithelial vs. non-epithelial—median (25th, 75th) Ct Ct zonulin*-18S*: *ztm* 19.96 (18.75, 20.91) vs. 17.10 (14.65, 18.92), *ztm-par2 −/−* 20.11 (18.51, 21.46) vs. 16.87 (15.27, 19.00), *ztm-*AT1001 22.30 (21.46, 23.27) vs. 19.19 (16.43, 20.95)] (Table 3). In the epithelial compartment of the stomach, *ztm-par2 −/−* had more zonulin expression than *ztm*-AT1001 mice (median (25th, 75th) median Ct zonulin*-18S* stomach: *ztm-par 2 −/−* 15.90 (15.17, 18.08) versus *ztm*-AT1001 21.66 (20.26, 22.25), Kruskal–Wallis test, *p* = 0.028) (Table 3). In the non-epithelial compartment, there was no difference in zonulin mRNA expression between mouse groups (Table 3). When examining expression between GI segments and within a mouse group in the epithelial compartment, zonulin mRNA expression was greater in the stomach compared to the jejunum in *ztm* and *ztm-par2 −/−* [median (25th, 75th) Ct zonulin*-18S*, stomach versus jejunum, *ztm* 18.01 (16.52, 18.60) versus 21.70 (20.91, 21.88), Kruskal–Wallis test *p* = 0.005, and *ztm-par2 −/−* 15.90 (15.17, 18.08) versus 24.29 (20.94, 24.55), Kruskal–Wallis test *p* = 0.048] (Table 3). In the non-epithelial compartment for all mouse groups, there was no difference in the zonulin mRNA expression among GI tissues within a mouse group (Table 3).

#### 2.4.2. Inflammatory Conditions

Median zonulin mRNA expression under inflammatory conditions for all mouse groups was greater in the non-epithelial compartment compared to the epithelial compartment [median (25th, 75th) Ct zonulin*-18S*, epithelial vs. non-epithelial: *ztm* 17.65 (15.87, 19.87) vs. 15.72 (12.73, 17.85), *ztm-par2-/-* 18.60 (18.30, 19.88) vs. 14.06 (13.35, 16.22), *ztm*-AT1001 19.19 (16.49, 21.11) vs. 15.19 (14.14, 16.56)] (Table 4). There was no difference in zonulin mRNA expression between mouse groups within GI segments in either compartment (Table 4). When examining expression between GI segments and within individual mouse groups in the epithelial compartment, *ztm* had more zonulin mRNA expression in the stomach and colon than the duodenum, [Ct zonulin*-18S:* stomach 14.29 (12.62, 17.56) versus duodenum 19.88 (18.97, 21.45), Kruskal–Wallis test *p* = 0.012 and duodenum 19.88 (18.97, 21.45) versus colon 15.64 (14.41, 18.25), Kruskal–Wallis test *p* = 0.016] (Table 4). In the non-epithelial compartment, *ztm-par2 -/-* mice had more zonulin mRNA expression in the stomach than the duodenum, [median (25th, 75th) Ct zonulin*-18S ztm-par 2-/- stomach* 11.54 (10.71, 14.15) versus duodenum 17.44 (16.36, 19.48), Kruskal–Wallis test *p* = 0.016] (Table 4).

### 2.5. par2 mRNA Expression in Epithelial Versus Non-Epithelial Compartment

*par2* mRNA expression was examined in the epithelial and non-epithelial compartments of all GI segments of zonulin- and *par2*-producing mouse groups, *ztm* and *ztm*-AT1001. Table 5 and Table 6 depict median (25th, 75th) Ct *par2-18S* mRNA expression in the epithelial and non-epithelial compartments of *ztm* and *ztm*-AT1001 under baseline and inflammatory conditions, respectively.

#### 2.5.1. Baseline Conditions

Median *par2* mRNA expression under baseline conditions was similar in the epithelial and non-epithelial compartments [median (25th, 75th) Ct *par2-18S* epithelial versus (vs) non-epithelial: *ztm* 13.46 (12.29, 15.03) vs. 14.16 (12.40, 16.92), *ztm-*AT1001 14.19 (12.88, 15.65) vs. 14.98 (14.06, 16.34)] (Table 5). Within the stomach, *ztm* had more *par2* expression than *ztm-*AT1001 [median (25th, 75th) Ct *par2-18S*: *ztm* 12.66 (11.79, 13.16) versus *ztm-*AT1001 13.86 (13.52, 13.98, Mann–Whitney U test *p* = 0.016] (Table 5). There was no difference in *par2* expression between *ztm* and *ztm*-AT1001 in the other GI segments. There was no difference in *par2* expression between GI segments for *ztm*. There was more *par2* mRNA expression in the ileum than the duodenum of *ztm*-AT1001, [median (25th, 75th) Ct *par2-18S*: ileum 12.30 (11.10, 12.66) versus duodenum 16.86 (15.45, 18.78), Kruskal–Wallis test, *p* = 0.012].

#### 2.5.2. Inflammatory Conditions

Median *par2* mRNA expression under inflammatory conditions was similar in the epithelial vs. non-epithelial compartments for *ztm* and *ztm-*AT1001 mice [median (25th, 75th) Ct *par2-18S*, epithelial vs. non-epithelial: *ztm* 13.17 (11.47, 16.02) vs. 14.45 (12.15, 16.87), *ztm-*AT1001 13.57 (12.24, 15.30) vs. 13.99 (12.87, 16.23)] (Table 6). There were no differences in *par2* expression between *ztm* and *ztm-*AT1001 in any of the GI segments in the epithelial and non-epithelial compartment (Table 6). The only difference between GI segments within mouse groups was noted in the epithelial compartment between the duodenum and colon of *ztm-*AT1001, [median (25th, 75th) Ct *par2-18S*, duodenum 15.38 (14.84, 17.80) versus colon 10.18 (8.50, 13.08), Kruskal–Wallis test *p* = 0.015] (Table 6).

## 3. Discussion

We have examined the relationship between zonulin and PAR2 on motility under inflammatory conditions, leveraging two models of zonulin inhibition and comprehensively examining gene expression in three tissue levels and all GI segments. We have demonstrated that under baseline conditions, motility follows similar patterns in zonulin expression and inhibition models to the (wt) control. However, triggering inflammation elicited distinct phenotypes, specifically with the *ztm* having faster small intestinal transit and zonulin inhibition reverting the phenotype back to the (wt) motility pattern. Differences in zonulin and *par2* mRNA expression were primarily noted when examining whole tissue between baseline and inflammatory conditions within mouse groups. Differences in zonulin and *par2* expression between mouse groups within GI segments and between GI segments within mouse groups in whole, epithelial and non-epithelial tissues were noted and support a complex and indirect role for zonulin on motility under conditions of inflammation.

An overall increase in zonulin under conditions of inflammation has been described for multiple diseases and in experimental models [14,16,17,27]. However, a comprehensive characterization of zonulin expression including multiple levels of GI tissue and segments, and in the context of motility, has not been described. In this study, *ztm* mice exhibited significantly increased zonulin expression after LPS injection across multiple GI segments, with the most pronounced upregulation observed in the stomach and proximal small intestine compared to baseline levels. This aligns with previous data reported on *ztm* by this group, including an increase in the zonulin mRNA expression of the stomach after LPS injection and an increase in zonulin expression in the duodenum and jejunum after dextran sodium sulfate (DSS) exposure [16,19]. It is this change in zonulin expression, as opposed to absolute zonulin expression, that drives the changes in motility after LPS injection, since the *ztm* had a similar motility pattern to (wt) mice under baseline conditions despite having zonulin expression and a different pattern of motility under conditions of inflammation when zonulin expression levels were increased in most GI segments. Furthermore, the GI segments with greatest dysmotility in the *ztm* were the segments with the greatest upregulation in zonulin expression. This is also supported by the mRNA expression for *par2* in the *ztm*. Zonulin acts via PAR2 and, therefore, differences in *par2* expression across GI segments could influence the effect of zonulin on GI function across tissues. However, *par2* mRNA expression was not different across GI segments under baseline or inflammatory conditions. The effect of zonulin on motility across GI tissues under conditions of inflammation, therefore, does not appear to be modulated by differences in *par2* itself, but rather the change in zonulin expression.

Although *par2* expression may not influence the differences in motility across GI segments in *ztm*, PAR2 is critical for the overall effect of zonulin. Both of our zonulin inhibition models block the PAR2 signaling pathway, *ztm-par2 −/−,* by a whole body receptor knockout and in *ztm*-AT1001 by the binding of a competitive peptide, AT1001, on PAR2 [12,13,26]. These two models of inhibition had dysmotility patterns more akin to the (wt) mouse after LPS injection, with minimal to no FITC reaching the distal small intestine, cecum or colon. This reversion to the (wt) motility phenotype in the inhibition models was present despite differences in the upregulation of zonulin, with increased expression in most GI segments for *ztm-par2 −/−* and some for *ztm-*AT1001. This confirms that inhibiting PAR2 blocked the effect of zonulin and its effect on motility, under conditions of inflammation. Previous models of intestinal inflammation have shown that AT1001 blocks the effect of zonulin, resulting in changes in GI physiology—specifically, an epithelial barrier leak [16]. No models of *ztm-par2 −/−*, however, have been previously reported and we noted differences between the two inhibition models regarding the degree of dysmotility and zonulin mRNA expression. The *ztm-par 2 −/−* had a dysmotility pattern that was almost identical to that of (wt) mice, whereas the *ztm-*AT1001 had an exaggerated dysmotility pattern after LPS injection. In effect, a mouse from the *ztm-par 2 −/−* group phenotypically resembles a (wt) mouse, since despite zonulin expression, it has no receptor for zonulin to affect at any time in its development and life cycle. However, the *ztm-*AT1001 mouse has zonulin and PAR2 throughout its development with the abrupt inhibition of this pathway, resulting in potential compensatory mechanisms that contributed to the exaggerated phenotype. The *ztm* has been described to have differences in its microbiome and immune cell composition compared to (wt) under baseline conditions [28]. Specifically, *ztm* has an overall pro-inflammatory microbial composition with a reduced abundance of *Akkermansia* and increased abundance of *Rikinella* bacterial species. Furthermore, it has a pro-inflammatory immune profile that is microbiome-independent, since alteration of the microbiome did not reverse this phenotype. The sudden inhibition of zonulin in *ztm* by the AT1001 peptide likely changes transepithelial trafficking of *ztm*’s altered microbiome which then impacts immune composition and, therefore, GI physiology. Future experiments will examine changes in the microbiome and immune profile to understand how the different approaches to zonulin inhibition impact GI physiology.

The role of PAR2 has been examined in various models of motility [23,24,25]. In a mouse model, intraperitoneal injection of PAR2 agonists resulted in faster transit, measured by the distance traversed of a marker within the small intestine [23]. Another study examined the role of PAR2 in motility after GI injury in a mouse model and demonstrated that PAR2 activation after injury accelerated transit compared to the sham [29]. The motility phenotype demonstrated in the GI injury model parallels that of our *ztm* model of inflammation, whereby *ztm* has attenuated dysmotility under conditions of inflammation compared to (wt)*,* but no difference in transit at the baseline despite *ztm* having zonulin expression and, therefore, PAR2 activation. Similarly, *par2* mRNA expression was different between *ztm* and *ztm*-AT1001 under baseline conditions when the GI motility phenotype was the same. These findings support an interaction between inflammation and PAR2 for PAR2 to have a role in motility. These findings also emphasize the importance of PAR2 as the receptor for zonulin to have its effect, but do not support an independent role for PAR2 on motility since absolute changes in PAR2 were not associated with the phenotype.

In summary, *ztm* has attenuated dysmotility, particularly in the small intestine. This appears to be due to changes in zonulin expression and zonulin activity on PAR2, as evidenced by the reversal of the phenotype in the zonulin inhibition models. Although zonulin and PAR2 are present in multiple cell populations across the GI tissue, our findings suggest that the effect of zonulin and PAR2 on motility is mediated primarily via the epithelial compartment. We identified few differences in zonulin and *par2* expression among mouse groups only in the epithelial compartment and under baseline conditions. However, effector cells that impact motility are in the non-epithelial compartment of the GI tissue (e.g., resident macrophages and enteric neurons), which supports an indirect role for zonulin on motility. As previously described, zonulin increases epithelial barrier leak, particularly under conditions of inflammation, and *ztm* has an altered microbiome [11,12,13,28]. An increase in transepithelial trafficking in *ztm* of intraluminal factors, including metabolites and microbial products, can impact downstream cell populations that regulate motility, whether under baseline or inflammatory conditions. Such cell populations include immune cells, particularly macrophages, and enteroendocrine cells [30,31]. Therefore, we postulate that zonulin impacts motility due to the differential priming of downstream cell populations under baseline conditions, which influences the inflammatory response of these cells. This relationship between transepithelial trafficking secondary to zonulin and motility is pertinent to inflammatory diseases where therapies to improve an epithelial barrier leak could negatively impact motility.

This study was unique in its use of two zonulin inhibition models which have not been widely studied. We also performed a comprehensive gene expression analysis of zonulin and *par2* in multiple tissue levels of the GI tract and within all GI segments. This allowed for an examination of the effect of zonulin and *par2* on motility at a segment-specific level and allowed for an exploration of how zonulin may modulate motility, whether directly or indirectly. Subsequent studies will examine in depth the potential indirect pathways by which transepithelial trafficking from an increase in zonulin mRNA expression may modulate dysmotility under conditions of inflammation. Furthermore, the regulation of motility, like many GI functions, is multifactorial and, therefore, more than one of its regulatory mechanisms were likely impacted under conditions of inflammation.

## 4. Materials and Methods

### 4.1. Animals

Experiments were performed in adult (8–12 week-old) male and female mice for all included genotypes and experimental groups. All mice were in a C57Bl/6 background and bred and maintained in an in-house colony at the Massachusetts General Hospital (MGH). Mice included (a) (wt) mice, which do not produce zonulin, as control, (b) *ztm*, which produce zonulin, (c) *ztm-par2 −/−*, which produce zonulin and lack the primary receptor for zonulin activity, PAR2, and (d) *ztm* exposed to AT1001, a zonulin inhibitor, so designated *ztm*-AT1001. The *ztm* was donated by Dr. Andrew Levy and its construct has been previously published [32]. The *ztm-par2 −/−* mice were generated by breeding *ztm* with commercially available *par2 −/−* mice. All mice were bred and housed under the same standard conditions per MGH regulations. Separate cohorts of mice were utilized for GI transit testing and for tissue isolation for mRNA expression. This animal protocol was approved by the MGH Institutional Animal Care and Use Committee (IACUC) and all procedures were performed in accordance with the MGH IACUC. All mice were euthanized per IACUC guidelines with isoflurane anesthetic overdose (5% isoflurane with secondary physical method). ARRIVE guidelines were followed. Weights were obtained 24–48 h prior to the experiment. All mouse experiments were run at a minimum in triplicate.

### 4.2. Experimental Conditions

#### 4.2.1. Inflammation

Inflammation was triggered by lipopolysaccharide (LPS) injection as previously published [33]. Mice were injected with 10 mg/kg of LPS or 1% Phosphate Buffered Saline (PBS) as vehicle control. The LPS utilized in these experiments was Escherichia Coli LPS O111:B4 (L3012, Sigma-Aldrich, St Louis, MO, USA), which was reconstituted in sterile 1% PBS. Mice were weighed 24–48 h prior to an experiment and injected 6 h prior to GI motility testing or euthanasia for tissue isolation [19]. We have previously shown this protocol to elicit significant inflammation with cytokine changes and the use of a sepsis score [19]. We confirmed inflammation in these experiments by the use of the sepsis score 6 h after injection [34]. Mice were excluded if they did not show signs and symptoms of inflammation by the sepsis score. Mice were fasted from standard chow at the time of injection and from water at the time of gavage. Mice within the same genotype were distributed randomly to be injected with vehicle control or LPS. Blinding from inflammatory conditions was not possible given the explicit changes in behavior as a result of LPS injection.

#### 4.2.2. Zonulin Inhibition by AT1001

AT1001 is an eight-amino-acid peptide, GGVLVQPG, that blocks zonulin activity by binding to PAR2. [26] AT1001 was produced by GenScript (Piscataway, NJ, USA) with a >98% purity. The *ztm* was exposed to 1 mg/mL of AT1001 in water for one week prior to completion of GI transit or tissue isolation. The AT1001 water was changed every other day and water intake (mL) was tracked. Animal weight was also tracked to ensure there were no adverse effects from AT1001.

### 4.3. Gastrointestinal Transit Testing

70 kDa of non-digestible fluorescein isothiocyanate-dextran (FITC-dextran) was purchased from MilliporeSigma, Burlington, MA, USA (MilliporeSigma FD70). Mice were gavaged with 6 mg/kg of FITC-dextran using a standard oral gavage needle 1 h prior to GI transit testing. GI tissue was isolated, including the stomach, small intestine, cecum and colon. Given the limitations of flushing the stomach and cecum, both tissues were cut open and then flushed with 2 mL of PBS. The small intestine was measured and divided into 10 equal segments; each segment was flushed with 2 mL of PBS. Lastly, the colon was flushed in total with 2 mL of PBS. The small intestine was further subdivided into the duodenum, jejunum, and ileum. Based on the average length of each segment, the first three (1–3) segments were determined to be the duodenum, the second three (4–6) segments, the jejunum and the last four (7–10) segments, the ileum. Each flush was diluted PBS at a 1:100 ratio and loaded in duplicates for measurement of FITC fluorescence using the Synergy-2 spectrophoto fluorimetry from BioTek, Santa Clara, CA, USA at 485/535 nm wavelengths. Given that the volume of FITC-dextran gavaged was dependent on individual mouse weight, FITC nanogram/mL measurements per GI segment were converted to percent based on total GI tract FITC measurement to allow for comparison among mouse groups and conditions.

### 4.4. Real-Time Quantitative-Polymerase Chain Reaction (q-PCR)

Stomach, duodenum, jejunum, ileum and colon tissue were isolated and processed whole and also divided into epithelial and non-epithelial compartments. The epithelial compartment was isolated from the non-epithelial compartment by scraping dissected tissue sections. All subsequent procedures were performed the same in whole tissue and epithelial versus non-epithelial tissue. The mRNA expression of zonulin and *par2* was examined by quantitative-Polymerase Chain Reaction (q-PCR), as previously published [19]. A 0.5 cm frozen piece of each segment, or scrape and residual tissue for the epithelial and non-epithelial compartments, from all mouse groups were collected and immediately placed in 1 mL of TRIzol^TM^ Reagent (Thermo Fisher, Waltham, MA, USA) and homogenized. mRNA was extracted using the Direct-zol RNA mini prep Kit (Zymo Research, Irvine, CA, USA) following manufacturer’s instructions. RNA concentrations and A260/A280 and A260/A230 ratios were measured with the NanoDrop spectrophotometer (Thermo Scientific, Waltham, MA, USA) to ensure the quality of the extracted mRNA. Target A260/A280 and A260/A230 ratios were ~2 and ~2–2.0. Any tissue that did not meet these criteria was discarded and mRNA extraction was performed with a new piece of tissue. Then, 1 µg of mRNA was used to perform the removal of genomic DNA, conversion to cDNA and the q-PCR. To ensure all genomic DNA was removed, we completed the DNAse step of the Direct-zol RNA mini prep kit and upon completion of mRNA extraction, also ran the DNA free^TM^ Kit (Thermo Fisher, Waltham, MA, USA) per manufacturer’s instructions. mRNA was reverse-transcribed using random hexamer primers and Maxima universal first-strand cDNA synthesis kit #1661 (Thermo Fisher Scientific, Waltham, MA, USA) according to manufacturers’ instructions.

Quantitative real-time PCR was performed using PerfeCTa SYBR^®^ Green SuperMix (Quantabio, Beverly, MA, USA) using in-house-designed primers based on NIH’s Primer Blast and obtained from Integrated DNA Technologies (Coralville, IA, USA) [*18S*-Primers 5′ Oligo AGAAACGGCTACCACATCCA 3′ Oligo CCCTCCAATGGATCCTCGTT, *zonuli*n-Primers 5′ Oligo GAATGTGAGGCAGATGACAG, 3′ Oligo GTGTTCACCCATTGCTTCTC; *par2*-Primers 5′ Oligo TCTGTCATCTGGTTCCCCCT, 3′ Oligo CGATCACCCAGTACCTCTGC]. For this, 1 uL (=1 µg) of cDNA was run per well and samples were run in duplicate; <20% difference between duplicates was ensured. If the difference was >20%, the samples were rerun. PerfeCTa SYBR^®^ Green SuperMix (Quantabio, Waltham, MA, USA) plus the primers but without the cDNA samples were run as blanks to ensure no contamination in our master mix. The delta cycle threshold (dCt), target gene Ct—housekeeping gene Ct, was calculated for all statistical analyses. The primary mRNA expression analysis was the difference between LPS and PBS conditions within each mouse group. Therefore, only Figure 3 and Figure 4 showing zonulin and *par2* mRNA expression under PBS and LPS conditions, show the 2^−ΔΔ*CT*^, where individual LPS dCt values were controlled for the mean dCt of PBS samples within the same mouse group. Controlling for the PBS mean dCt would not apply to subsequent analyses and, therefore, 2^−ΔΔ*CT*^ figures would misrepresent the relationship between data. Therefore, all secondary analyses considering differences between mouse groups or between GI tissues within mouse groups were performed using the dCt data and are represented in tabular format (Table 3, Table 4, Table 5 and Table 6). 

### 4.5. Statistical Analysis

Motility data were analyzed using R version 4.2.1 by the R Foundation for Statistical Computing (Vienna, Austria). Motility data are presented as mean and standard deviation. q-PCR statistical analyses were completed using GraphPad Prism Version 8, GraphPad Software La Jolla, California. In the setting of large biological variability, q-PCR data are presented as median (25th, 75th).

Male and female mice were analyzed in aggregate. Sample size was calculated based on previous mouse GI transit data and to identify a minimum 10% difference in GI transit between our primary experimental group, LPS-injected *ztm* and control with 80% power. A minimum sample size of 8 mice per study group, under baseline and inflammatory conditions, was selected to mitigate the inherent variance of the assay. GI transit between mouse groups was analyzed using Dirichlet regression. The outcome—FITC percentage across five intestinal segments (duodenum, jejunum, ileum, cecum and colon)—is compositional and adds up to 100%. Using a Dirichlet regression, all parts of the composition are modeled together, which preserves the internal correlation structure inherent in such datasets. Mouse groups were treated as independent variables, with the (wt) group as the reference and the distribution of FITC across the segments as the dependent variables (Figure 1 and Figure 2). Results are presented as odds ratio and 95% confidence interval (Supplementary Tables S1 and S2). All qPCR data were analyzed using Mann–Whitney U test or Kruskal–Wallis test, as applicable (Figure 3 and Figure 4, Table 1, Table 2, Table 3, Table 4, Table 5 and Table 6). All data from mice that exhibited expected inflammation post-LPS injection were included.

## 5. Conclusions

In the *ztm* model, we have demonstrated that changes in zonulin expression under conditions of inflammation are associated with distinct motility patterns and that PAR2 is necessary for zonulin to have its effect on motility. Zonulin and PAR2 within the epithelial compartment appear to be driving these differences in the phenotype, supporting a complex and indirect relationship between zonulin and downstream effector cell populations that modulate motility.

## Figures and Tables

**Table 1:** zonulin mRNA expression in whole tissue of GI segments.

Condition	Control			*p*-Value, Kruskal–Wallis Test Between Mouse Groups Within GI Segment *	Condition	LPS			*p*-Value, Kruskal–Wallis Test Between Mouse Groups Within GI Segment *
Mouse Group	*ztm*	*ztm-* *par2 −/−*	*ztm-*AT1001		Mouse Group	*ztm*	*ztm-* *par2 −/−*	*ztm-*AT1001	
GI Segment	Stomach					Stomach			
Median Ct	17.70	15.46	13.23	NS	Median Ct	9.90	13.14	13.66	NS
IQR	12.80, 17.46	14.68, 21.47	10.90, 14.87		IQR	8.77, 12.28	11.14, 13.85	8.85, 15.98	
GI Segment	Duodenum					Duodenum			
Median Ct	20.26	19.88	18.10	NS	Median Ct	15.61	17.68	14.89	NS
IQR	15.95, 21.17	19.08, 21.06	15.33, 19.54		IQR	12.07, 16.26	16.89, 18.52	13.71, 17.19	
GI Segment	Jejunum					Jejunum			
Median Ct	18.36	18.52	14.23	Z vs. ZA, *p* = 0.021	Median Ct	14.62	14.26	15.68	NS
IQR	15.99, 21.52	16, 20.98	12.07, 16.23		IQR	12.96, 15.79	13.44, 18.21	11.30, 17.08	
GI Segment	Ileum					Ileum			
Median Ct	14.77	17.59	16.01	Z vs. ZP, *p* = 0.013	Median Ct	12.04	13.68	14.73	NS
IQR	13.12, 15.49	16.33, 18.08	14.71, 18.83		IQR	10.16, 14.02	11.01, 15.46	8.55, 17.08	
GI Segment	Colon					Colon			
Median Ct	16.23	17.23	15.54	NS	Median Ct	14.01	12.88	11.82	NS
IQR	15.13, 19.86	16.23, 18.08	15.10, 19.87		IQR	11.45, 14.65	11.15, 14.72	11.12, 12.94	
*p*-value, Kruskal–Wallis test between GI segments within mouse group *	Duodenum vs. Ileum, *p* = 0.042	NS	Stomach vs. Duodenum, *p* = 0.0081		*p*-value, Kruskal–Wallis test between GI segments within mouse group *	NS	Duodenum vs. Colon, *p* = 0.041	NS	

* Corrected for multiple comparisons of GI segments within same mouse group, NS *p*-value > 0.05. Ct: cycle threshold; IQR: interquartile range represented as 25th and 75th percentile; GI: gastrointestinal; LPS: lipopolysaccharide; n: sample size; Z, *ztm*: zonulin transgenic mice; ZP, *ztm-par2 −/−*: *ztm*-protease-activated receptor 2 knockout; ZA, *ztm*-AT1001: *ztm* mice exposed to a zonulin inhibitor (AT1001) for 1 week preceding experiments; control is PBS (phosphate buffered saline)-injected mice.

**Table 2:** *par2* mRNA expression in whole tissue of GI segments.

Condition	Control		*p*-Value, Mann–Whitney U Test Between Mouse Groups Within GI Segment *	Condition	LPS		*p*-Value, Mann–Whitney U Test Between Mouse Groups Within GI Segment *
Mouse Group	*ztm*	*ztm-*AT1001		Mouse Group	*ztm*	*ztm-*AT1001	
GI Segment	Stomach			GI Segment	Stomach		
Median Ct	12.50	11.26	NS	Median Ct	13.43	12.34	NS
IQR	10.60, 15.02	10.79, 12.00		IQR	11.42, 13.72	10.56, 15.32	
GI Segment	Duodenum			GI Segment	Duodenum		
Median Ct	11.58	13.43	NS	Median Ct	13.11	16.00	NS
IQR	8.45, 13.86	12.38, 16.46		IQR	11.61, 14.68	12.36, 17.52	
GI Segment	Jejunum			GI Segment	Jejunum		
Median Ct	12.47	10.10	*p* = 0.011	Median Ct	12.87	11.49	NS
IQR	12.09, 14.01	9.74, 11.19		IQR	11.52, 16.59	11.09, 12.57	
GI Segment	Ileum			GI Segment	Ileum		
Median Ct	10.28	17.59	*p* = 0.008	Median Ct	12.80	16.71	*p* = 0.0022
IQR	8.99, 11.52	15.69, 18.63		IQR	11.88, 15.17	16.41, 17.42	
GI Segment	Colon			GI Segment	Colon		
Median Ct	8.92	16.16	*p* = 0.016	Median Ct	10.16	16.07	*p* = 0.004
IQR	8.66, 11.56	15.74, 16.56		IQR	9.20, 11.35	14.81, 17.27	
*p*-value, Kruskal–Wallis test between GI segments within mouse group *	NS	Duodenum vs. Jejunum, *p* = 0.023 Jejunum vs. Ileum, *p* = 0.0015 Jejunum vs. Colon, *p* = 0.018		*p*-value, Kruskal–Wallis test between GI segments within mouse group *	NS	Jejunum vs. Ileum, *p* = 0.0046 Jejunum vs. Colon, *p* = 0.046	

* Corrected for multiple comparisons of GI segments within same mouse group, NS *p*-value > 0.05. Ct: cycle threshold; IQR: interquartile range represented as 25th and 75th percentile; GI: gastrointestinal; LPS: lipopolysaccharide; n: sample size; *ztm*: zonulin transgenic mice; *ztm*-AT1001: *ztm* mice exposed to a zonulin inhibitor (AT1001) for 1 week preceding experiments; control is PBS (phosphate buffered saline)-injected mice.

**Table 3:** zonulin mRNA expression in PBS-injected mouse groups across the epithelial and non-epithelial compartments of GI segments.

Condition	Epithelial Compartment			*p*-Value, Kruskal–Wallis Test Between Mouse Groups Within GI Segment *	Condition	Non-Epithelial Compartment			*p*-Value, Kruskal–Wallis Test Between Mouse Groups Within GI Segment *
Mouse Group	*ztm*	*ztm-* *par2 −/−*	*ztm-*AT1001		Mouse Group	*ztm*	*ztm-* *par2 −/−*	*ztm-*AT1001	
GI Segment	Stomach				GI Segment	Stomach			
Median Ct	18.01	15.90	21.66	ZP vs. ZA, *p* = 0.028	Median Ct	16.15	15.03	18.17	NS
IQR	16.52, 18.60	15.17,18.08	20.26, 22.25		IQR	13.81, 19.22	10.88, 16.41	16.63, 19.33	
n	5	3	4		n	7	4	4	
GI Segment	Duodenum				GI Segment	Duodenum			
Median Ct	19.97	22.05	21.42	NS	Median Ct	17.73	19.11	19.29	NS
IQR	18.46, 22.69	21.43, 22.68	21.11, 22.37		IQR	15.50, 20.54	18.47,19.74	17.64, 20.97	
n	4	2	4		n	6	2	3	
GI Segment	Jejunum				GI Segment	Jejunum			
Median Ct	21.70	24.29	23.11	NS	Median Ct	18.03	17.26	17.89	NS
IQR	20.91, 21.88	20.94, 24.55	23.02, 23.92		IQR	14.98, 18.99	15.95, 20.31	15.36, 21.55	
n	7	5	3		n	7	3	4	
GI Segment	Ileum				GI Segment	Ileum			
Median Ct	20.13	20.06	23.00	NS	Median Ct	18.25	16.15	20.99	NS
IQR	18.19, 21.06	17.84, 22.28	21.47, 24.43		IQR	15.17, 19.90	15.44, 19.83	17.60, 22.52	
n	9	2	4		n	6	4	4	
GI Segment	Colon				GI Segment	Colon			
Median Ct	19.97	18.23	22.29	NS	Median Ct	15.36	16.81	19.60	NS
IQR	19.65, 20.30	17.15, 19.72	21.22, 23.36		IQR	13.77, 15.94	15.62, 18.70	14.94, 20.40	
n	2	4	2		n	3	4	4	
*p*-value, Kruskal–Wallis test between GI segments within mouse group *	Stomach vs. Jejunum, *p* = 0.005	Stomach vs. Jejunum, *p* = 0.048	NS		*p*-value, Kruskal–Wallis test between GI segments within mouse group *	NS	NS	NS	

* Corrected for multiple comparisons of GI segments within same mouse group, NS *p*-value > 0.05. Ct: cycle threshold; IQR: interquartile range represented as 25th and 75th percentile; GI: gastrointestinal; LPS: lipopolysaccharide; n: sample size; Z, *ztm*: zonulin transgenic mice; ZP, *ztm-par2 −/−*: *ztm*-protease-activated receptor 2 knockout; ZA, ztm-AT1001: *ztm* mice exposed to a zonulin inhibitor (AT1001) for 1 week preceding experiments; control is PBS (phosphate buffered saline)-injected mice.

**Table 4:** zonulin mRNA expression in LPS-injected mouse groups across the epithelial and non-epithelial compartments of GI segments.

Condition	Epithelial Compartment			*p*-Value, Kruskal–Wallis Test Between Mouse Groups Within GI Segment *	Condition	Non-Epithelial Compartment			*p*-Value, Kruskal–Wallis Test Between Mouse Groups Within GI Segment *
Mouse Group	*ztm*	*ztm-* *par2 −/−*	*ztm-AT1001*		Mouse Group	*ztm*	*ztm-* *par2 −/−*	*ztm-AT1001*	
GI Segment	Stomach				GI Segment	Stomach			
Median Ct	14.29	13.68	18.08	NS	Median Ct	14.08	11.54	13.09	NS
IQR	12.62, 17.56	13.51,17.19	15.03, 19.80		IQR	11.87, 16.07	10.71, 14.15	12.79, 14.65	
n	5	3	5		n	6	5	5	
GI Segment	Duodenum				GI Segment	Duodenum			
Median Ct	19.88	20.93	20.23	NS	Median Ct	17.90	17.44	16.62	NS
IQR	18.97, 21.45	19.60, 23.82	19.88, 22.64		IQR	14.34, 20.27	16.36,19.48	16.18, 17.06	
n	10	4	5		n	5	4	2	
GI Segment	Jejunum				GI Segment	Jejunum			
Median Ct	19.70	22.79	21.02	NS	Median Ct	15.00	14.69	15.69	NS
IQR	17.49, 21.30	22.79, 22.79	17.37, 22.43		IQR	11.15, 16.59	14.16, 16.67	14.15, 17.18	
n	9	1	5		n	6	3	5	
GI Segment	Ileum				GI Segment	Ileum			
Median Ct	18.76	17.96	18.31	NS	Median Ct	15.91	13.04	16.44	NS
IQR	15.86, 20.79	17.96, 17.96	12.64, 20.71		IQR	13.74, 17.92	12.35, 16.55	14.06, 17.82	
n	7	1	4		n	4	5	5	
GI Segment	Colon				GI Segment	Colon			
Median Ct	15.64	17.65	18.29	NS	Median Ct	15.73	13.60	14.09	NS
IQR	14.41, 18.25	17.65, 17.65	17.54, 19.97		IQR	12.57, 18.41	13.15, 14.23	13.50, 16.09	
n	9	1	5		n	4	5	5	
*p*-value, Kruskal–Wallis test between GI segments within mouse group *	Stomach vs. Duodenum, *p* = 0.012 Duodenum vs. Colon, *p* = 0.016	NS	NS		*p*-value, Kruskal–Wallis test between GI segments within mouse group *	NS	Stomach vs. Duodenum, *p* = 0.016	NS	

* Corrected for multiple comparisons of GI segments within same mouse group, NS *p*-value > 0.05. Ct: cycle threshold; IQR: interquartile range represented as 25th and 75th percentile; GI: gastrointestinal; LPS: lipopolysaccharide; n: sample size; Z, *ztm*: zonulin transgenic mice; ZP, *ztm-par2 −/−*: *ztm*-protease-activated receptor 2 knockout; ZA, *ztm*-AT1001: *ztm* mice exposed to a zonulin inhibitor (AT1001) for 1 week preceding experiments; control is PBS (phosphate buffered saline)-injected mice.

**Table 5:** *par2* mRNA expression in PBS-injected mouse groups across the epithelial and non-epithelial compartments of GI segments.

Condition	Epithelial Compartment		*p*-Value, Mann–Whitney U Test Between Mouse Groups Within GI Segment *	Condition	Non-Epithelial Compartment		*p*-Value, Mann–Whitney U Test Between Mouse Groups Within GI Segment *
Mouse Group	*ztm*	*ztm-*AT1001		Mouse Group	*ztm*	*ztm-*AT1001	
GI Segment	Stomach			GI Segment	Stomach		
Median Ct	12.66	13.86	*p* = 0.016	Median Ct	13.99	13.79	NS
IQR	11.79, 13.16	13.52, 13.98		IQR	12.73, 17.23	12.91, 14.66	
n	5	4		n	7	4	
GI Segment	Duodenum			GI Segment	Duodenum		
Median Ct	14.20	16.86	NS	Median Ct	16.64	16.69	NS
IQR	12.79, 16.21	15.45, 18.78		IQR	15.05, 19.79	16.31, 17.78	
n	6	4		n	6	3	
GI Segment	Jejunum			GI Segment	Jejunum		
Median Ct	15.54	14.77	NS	Median Ct	12.62	13.97	NS
IQR	14.11, 16.35	12.82, 17.99		IQR	11.15, 12.80	12.82, 15.75	
n	9	4		n	7	4	
GI Segment	Ileum			GI Segment	Ileum		
Median Ct	11.84	12.30	NS	Median Ct	16.47	19.38	NS
IQR	11.25, 14.27	11.10, 12.66		IQR	13.45, 19.54	17.27, 20.48	
n	10	4		n	6	4	
GI Segment	Colon			GI Segment	Colon		
Median Ct	13.04	13.17	NS	Median Ct	11.07	11.08	NS
IQR	11.51, 15.16	11.51, 14.84		IQR	9.60, 15.23	10.98, 13.04	
n	3	2		n	3	4	
*p*-value, Kruskal–Wallis test between GI segments within mouse group *	NS	Duodenum vs. Ileum, *p* = 0.012		*p*-value, Kruskal–Wallis test between GI segments within mouse group *	Duodenum vs. Jejunum, *p* = 0.026	NS	

* Corrected for multiple comparisons of GI segments within same mouse group, NS *p*-value > 0.05. Ct: cycle threshold; IQR: interquartile range represented as 25th and 75th percentile; GI: gastrointestinal; LPS: lipopolysaccharide; n: sample size; Z, *ztm*: zonulin transgenic mice; ZA, ztm-AT1001: *ztm* mice exposed to a zonulin inhibitor (AT1001) for 1 week preceding experiments; control is PBS (phosphate buffered saline)-injected mice.

**Table 6:** *par2* mRNA expression in LPS-injected mouse groups across the epithelial and non-epithelial compartments of GI segments.

Table	Epithelial Compartment		*p*-Value, Mann–Whitney U Test Between Mouse Groups Within GI Segment *	Condition	Non-Epithelial Compartment		*p*-Value, Mann–Whitney U Test Between Mouse Groups Within GI Segment *
Mouse Group	*ztm*	*ztm-*AT1001		Mouse Group	*ztm*	*ztm-*AT1001	
GI Segment	Stomach			GI Segment	Stomach		
Median Ct	13.07	13.41	NS	Median Ct	14.89	14.12	NS
IQR	11.76, 13.45	12.95, 14.28		IQR	11.36, 19.02	13.88, 17.35	
n	5	5		n	6	5	
GI Segment	Duodenum			GI Segment	Duodenum		
Median Ct	12.98	15.38	NS	Median Ct	14.74	14.93	NS
IQR	12.30, 16.27	14.84, 17.80		IQR	12.28, 16.69	13.16, 16.70	
n	10	5		n	3	2	
GI Segment	Jejunum			GI Segment	Jejunum		
Median Ct	16.16	16.28	NS	Median Ct	13.00	13.43	NS
IQR	13.22, 17.19	13.27, 17.41		IQR	12.01, 13.88	12.41, 15.53	
n	9	5		n	6	5	
GI Segment	Ileum			GI Segment	Ileum		
Median Ct	11.15	12.60	NS	Median Ct	16.13	15.71	NS
IQR	10.38, 18.94	11.64, 13.95		IQR	12.91, 18.12	13.81, 18.07	
n	6	4		n	5	5	
GI Segment	Colon			GI Segment	Colon		
Median Ct	12.51	10.18	NS	Median Ct	13.50	11.75	NS
IQR	9.70, 14.24	8.50, 13.08		IQR	12.20, 16.63	11.11, 13.50	
n	9	5		n	4	5	
*p*-value, Kruskal–Wallis test between GI segments within mouse group *	NS	Duodenum vs. Colon, *p* = 0.015		*p*-value, Kruskal–Wallis test between GI segments within mouse group *	NS	NS	

* Corrected for multiple comparisons of GI segments within same mouse group, NS *p*-value > 0.05. Ct: cycle threshold; IQR: interquartile range represented as 25th and 75th percentile; GI: gastrointestinal; LPS: lipopolysaccharide; n: sample size; Z, *ztm*: zonulin transgenic mice; ZA, *ztm*-AT1001: *ztm* mice exposed to a zonulin inhibitor (AT1001) for 1 week preceding experiments; control is PBS (phosphate buffered saline)-injected mice.

## Data Availability

The original data presented in the study are openly available in Harvard Dataverse at https://doi.org/10.7910/DVN/CJXYGL.

## Supplementary Materials

The following supporting information can be downloaded at: https://www.mdpi.com/article/10.3390/ijms26136381/s1.

## Abbreviations

Fluorescein–isothiocyanate, FITC; gastrointestinal, GI; lipopolysaccharide, LPS; protease-activated receptor 2, PAR2; quantitative-polymerase chain reaction, qPCR; zonulin transgenic mouse, *ztm*; *ztm-par2* knockout, *ztm-par2 −/−*; ztm and AT1001 exposed, *ztm*-AT1001; wildtype, (wt)*;* zonulin and *par2* are presented in lowercase and italics when when referring to mRNA or the mouse models.
