# Supplementary material for: Characterizing zonulin and par2 Expression in Zonulin Transgenic and Zonulin Inhibition Mouse Models of Motility and Inflammation
Source: Int J Mol Sci. 2025 Jul 2;26(13):6381. doi: 10.3390/ijms26136381 (PMC12249515; doi:10.3390/ijms26136381)
Supplement: Supplementary file 1 [file ijms-26-06381-s001.zip › ijms-3566759-supplementary.pdf]

Supplemental Table S1. Dirichlet Regression of %FITC in all gastrointestinal segments under baseline conditions in *zonulin* expressing mouse models controlled for *wildtype* mice

GI Segment	Mouse Group	OR	95% CI	p-value
Stomach	*ztm*	0.62	(0.25, 1.51)	0.291
	ztm-par2 -/-	1.17	(0.52, 2.62)	0.704
	ztm-AT1001	1.55	(0.6, 4.06)	0.368
Duodenum	*ztm*	0.77	(0.31, 1.89)	0.571
	ztm-par2 -/-	1.43	(0.64, 3.21)	0.382
	ztm-AT1001	1.41	(0.54, 3.71)	0.482
Jejunum	*ztm*	0.4	(0.17, 0.97)	0.043
	ztm-par2 -/-	0.81	(0.37, 1.79)	0.603
	ztm-AT1001	0.78	(0.3, 2.01)	0.601
Ileum	*ztm*	0.48	(0.2, 1.15)	0.098
	ztm-par2 -/-	1.51	(0.71, 3.18)	0.283
	ztm-AT1001	0.5	(0.2, 1.27)	0.143
Colon	*ztm*	0.95	(0.39, 2.32)	0.914
	ztm-par2 -/-	3.26	(1.5, 7.11)	0.003
	ztm-AT1001	0.6	(0.23, 1.58)	0.301

wildtype n= 11; *ztm*: zonulin transgenic mice, n=8; *ztm-par2 -/-* : ztm protease activated receptor 2 knockout, n=11; *ztm*-AT1001: ztm exposed to AT1001, a zonulin inhibitor, n=6

Supplemental Table S2. Dirichlet Regression of %FITC in all gastrointestinal segments under conditions of inflammation in *zonulin* expressing mouse models controlled for *wildtype* mice

GI Segment	Mouse Group	OR	95% CI	p-value
Stomach	*ztm*	0.52	(0.24, 1.16)	0.109
	ztm-par2 -/-	0.89	(0.43, 1.86)	0.761
	ztm-AT1001	3.16	(1.66, 5.99)	<0.001
Duodenum	*ztm*	0.61	(0.27, 1.38)	0.236
	ztm-par2 -/-	0.91	(0.43, 1.93)	0.803
	ztm-AT1001	5.75	(3, 10.99)	<0.001
Jejunum	*ztm*	0.24	(0.11, 0.54)	0.001
	ztm-par2 -/-	0.55	(0.26, 1.17)	0.12
	ztm-AT1001	0.57	(0.29, 1.12)	0.102
Ileum	*ztm*	0.84	(0.37, 1.94)	0.689
	ztm-par2 -/-	1.13	(0.51, 2.48)	0.767
	ztm-AT1001	1.59	(0.78, 3.24)	0.205
Colon	*ztm*	1	(0.43, 2.31)	0.999
	ztm-par2 -/-	1.17	(0.52, 2.61)	0.705
	ztm-AT1001	1.92	(0.93, 3.96)	0.078

wildtype n= 15; *ztm*: zonulin transgenic mice, n=8; *ztm-par2 -/-* : ztm protease activated receptor 2 knockout, n=9; *ztm*-AT1001: ztm exposed to AT1001, a zonulin inhibitor, n=12
